# Tumor Necrosis Factor-Alpha in Peripical Tissue Exudates of Teeth with Apical Periodontitis

**DOI:** 10.1155/2007/69416

**Published:** 2008-01-24

**Authors:** Sonja Pezelj-Ribarić, Karolina Magašić, Jelena Prpić, Ivana Miletić, Zoran Karlović

**Affiliations:** ^1^1Department of Oral Medicine, Medical Faculty, University of Rijeka, Brace Branchetta 20, 51000 Rijeka, Croatia; ^2^2Department of Endodontology and Restorative Dentistry, University of Zagreb, Trg maršala Tita 14, 10000 Zagreb, Croatia

## Abstract

*Aim.* The aim of this study was to determine tumor necrosis factor-alpha (TNF-α) levels in periapical exudates and to evaluate their relationship with radiological findings. *Methodology.* Periapical exudates were collected from root canals of 60 single-rooted teeth using absorbent paper points. TNF-α levels were determined by enzyme-linked immunosorbent assays. The samples were divided into three groups according to the periapical radiolucent area. *Results.* Nonparametric Kruskal-Wallis test revealed significant differences between TNF-α concentrations in control group (40, 57±28, 15 pg/mL) and group with larger radiolucent areas (2365, 79±582, 95 pg/mL), as well as between control and canals with small radiolucent areas (507, 66±278, 97) (P<.05). *Conclusions.* The levels of TNF-α increase significantly in teeth with periapical pathosis, from smaller to bigger lesions. This research and its results have shown that objective analysis of the TNF-α levels enables establishment of a relationship between different concentrations of TNF-α and different radiological changes.

## 1. INTRODUCTION

Periapical pathoses of pulpal origin develop in response to microbial irritants in the root canal systems. Bacterial cell wall components react with monocytes, macrophages, other cells of the immune system, as well as with fibroblasts, leading to the production of proinflammatory cytokines, such as IL-1*α*, IL-1*β*, TNF-*α*, IL-6, and IL-8 [1].

Persistent injuries to the dental pulp usually cause irreversible pulpitis and pulpal necrosis. Irritants may be mechanical or chemical, but are most often bacterial. The interaction between these irritants and host defensive cells results in release of numerous mediators that, through the root canal system, are capable of initiating immunologic reactions in periapical tissues resulting in the formation on inflammatory periapical lesions. These reactions include immune response mediated by cells, through the actions of T lymphocytes and cytokines, and hummoral immune response mediated by antibodies, activated by B-lymphocyte products [2].

TNF-*α* is a monocyte-derived protein that has a wide range of proinflammatory and immunomodulatory effects on a number of different cell populations. TNF-*α* is a cytokine which stimulates bone resorption, prostaglandin synthesis, and protease production by many cell types, including fibroblasts and osteoblasts. Overproduction or inappropriate expression of TNF-*α* can lead to a variety of pathological conditions [3]. The local production of PGE_2_, IL1-*β*, and TNF-*α* has been demonstrated in periapical lesions [4, 5].

TNF-*α* is a cytokine initially identified as a causative of hemorrhagic necrosis in certain tumors and was later shown to be the same molecule as cachectin, a serum product earlier known as a mediator of wasting in chronic disease. In addition to its diverse bioactivities, TNF-*α* is the only molecule other than IL-1 that is presently known to have osteoclast-activating function. Although extensive research has been performed in the area of periapical inflammatory mediators, only few studies focused on the role and levels of TNF-*α* in periapical exudate [6, 7]. The aim of this study was to determine the levels of TNF-*α* in periapical exudates and to evaluate possible relationships between this cytokine and radiological findings in the involved teeth.

## 2. MATERIALS AND METHODS

The investigation was carried out on 60 subjects of different age and sex. Subjects were divided into three groups according to the radiological findings. Diagnoses were established according to the clinical and radiological findings. All patients in the certain period were included. The patients with noncontributory medical histories and diagnosed with apical periodontitis were selected for the study as follows.

- Group 1 consisted of 20 subjects whose single-rooted teeth had the diagnosis of chronic apical periodontitis, with dull or absent pain, and size of the lesion <1 cm.
- Group 2 consisted of 20 subjects whose single-rooted teeth were diagnosed with chronic apical periodontitis, with dull or absent pain, but with the lesion size of >1 cm.
- Group 3 was control and comprised of 20 subjects whose investigated single-rooted teeth showed symptoms of acute periapical condition accompanied by excruciating pain, necrotic pulp, and radiological thickening of the apical periodontal ligament.

Primary access to the pulp chamber was gained using slow-speed round dental burs, with placement of rubber dam isolation. After determination of the working length, the necrotic pulp was removed and the root canal was enlarged to ISO size 40. The root canal was dried with sterilized paper points. A sterile paper point was inserted into the root canal up to the instrumentation length and held in place for 1 minute to absorb the exudate for the sample [6, 7]. Paper points were immersed into sterile Ependorf vials containing phosphate buffered saline (PBS) and stored at ${-70}^{\circ }$C until further analysis [8, 9]. The volume of the fluid was calculated from a standard curve and expressed as μL. Size of the radiolucent area was determined by two independent examiners at the time exudate samples were collected. Periapical lesions were classified as small, when the longer diameter was shorter than 1 cm, while the remaining lesions were classified as large. In the control group, the radiological thickening of the periodontal ligament was present.

Determination of the TNF-*α* levels in supernatant of periapical exudate is based on utilization of monoclonal antibodies specific for TNF-*α* which bind competitively to TNF-*α*. Investigation is based on “sandwich” enzyme immunoassay (EIA).

All subjects were informed of the aims and procedures of research. Within the research, they are guaranteed in respect of their basic ethical and bioethical principles: personal integrity (independence, righteousness, well-being, and safety) as regulated by Nűrnberg codex and the most recent version of Helsinki declaration. Only those subjects who gave a written permission in the form of informed consent were included.

## 3. RESULTS

**Figure 1:**
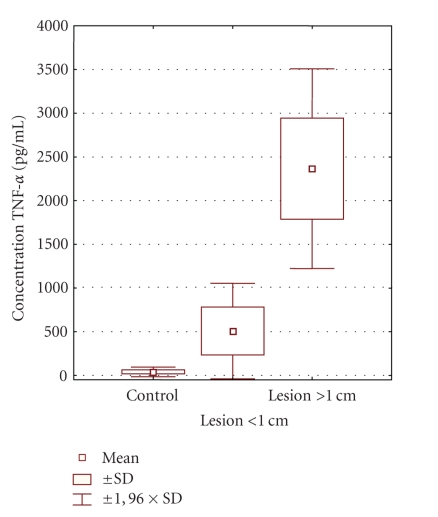
Levels of cytokine TNF-α in three groups of investigated subjects.

The present study demonstrated the presence of TNF-*α* in all periapical exudate samples. Differences in concentrations of TNF-*α* between control group (40, 57 ± 28, 15 pg/mL), the group with large radiolucent areas (2365, 79 ± 582, 95 pg/mL), as well as between control and the canals associated with small radiolucent areas (507, 66 ± 278, 97) were statistically significant (*P* < .05). There was also statistically significant difference between the samples with large radiolucent areas and small radiolucent areas. EIA data for TNF-*α* were categorized according to radiological diagnosis, using the nonparametric Kruskal-Wallis test (see Figure 1).

## 4. DISCUSSION

Soluble mediators produced and secreted by various inflammatory, immunologically-active, and structural cells, commonly referred to as cytokines, play the leading role in pathogenesis of infectious disease, acting side-by-side with other inflammatory mediators: kinines, vasoactive amines, components of the complement system, and metabolites of arachidonic acid [10].

Secretion of the cytokines is initiated with the purpose of activating immunological response to irritants and increasing local concentrations of inflammatory cells in order to prevent further colonization of bacteria within the tissues. Enhanced reaction of the host to various antigens results in bone resorption and formation of granulomatous tissue, which are the typical features of periapical lesion [10].

In this investigation, levels of the proinflammatory cytokine TNF-*α* in periapical exudate were analyzed due to its leading role in pathogenesis of periapical lesions. Exudates were collected from the root canals using noninvasive methods. These exudates present the inflammatory exudate from the periapical tissues and theoretically contain locally produced and secreted factors [11]. They resulted from the inflammatory response and contain host mediators related in response to the infection [6]. Highest concentrations of TNF-*α* were detected in periapical lesions with large radiolucent areas, while these levels were significantly lower in the periapical lesions with smaller radiolucent areas. In the control group diagnosed as acute periodontitis, with radiological changes consistent with periodontal ligament thickening, which differs in clinical symptoms from the other two experimental groups, and is characterized by radioloical changes consistent with periodontal ligament thickening, the levels of the investigated cytokine demonstrated the lowest levels. These results show different values from the results obtained by Ataoglu et al. [6]; however, these authors compared the levels of two kinds of cytokines: IL-1*ß* and TNF-*α*. In the work of Ataoglu et al. [6], IL-1*ß* was detected in all samples of periapical exudates and its levels were significantly higher than TNF-*α* levels; however, the authors focused their attention only on the TNF-*α* levels in periapical exudates demonstrated in all tissue specimens with significant differences regarding certain experimental groups. A study by Kjeldsen et al. [12] using ELISA technique also showed significantly higher concentrations of TNF-*α* in crevicular fluid in patients with chronic adult periodontitis. It is likely that chronic periodontal infection may evoke an immune response that may result in the production of slightly higher levels of TNF-*α*.

It was proved that TNF-*α* leads to bone resorption through osteoclast activation and stimulation of the secretion of proteolytic enzymes, plasminogen activator (PA), and matrix metalloproteinases (MMP), which are in charge of destroying extracellular matrix of the bone tissue [13].

The work by Artese et al. [14] showed that there is a small fraction of TNF-*α*-positive cells within the granuloma with a macrophage-like morphology. Ultrastructural analyses showed that there are some macrophages which have adjusted the extracellular secretion; therefore, these macrophages might be the main source of cytokines in the tissue.

Wang et al. [15] showed that both TNF-*α* and IL-1 are secreted in the infected rat pulps and periapical lesions. The cells appeared as soon as 2 days following the pulp chamber opening and their numbers increased steadily until the day 30. These findings demonstrate the presence of IL-1*α*- and TNF-*α*-secreting cells in the pulp and periapical tissues immediately following the pulp exposure, which supports the assumption that the abovementioned cytokines play a role in pathogenesis of pulp and periapical pathoses.

In the analysis of TNF-*α* levels in healthy, symptomatic, and asymptomatic human pulps [16], TNF-*α* was detected in all vital pulp tissues. The highest concentrations of TNF-*α*, with regard to clinical classification, were found in symptomatic reversible pulpitis, and the difference was statistically significant in comparison with irreversible asymptomatic pulpitis and healthy pulp. The highest concentrations of TNF-*α*, with regard to histological classification, were found in histologically confirmed moderate inflammation, followed by severe inflammation, mild inflammation, and finally healthy pulp.

## 5. CONCLUSION

Conclusion can be drawn that cytokine TNF-*α* could be an objective marker in radiologically confirmed periapical lesions. Presence of TNF-*α* in this investigation was proved in all clinical samples. The lowest levels were demonstrated in control group, which was characterized by radiological thickening of the periodontal ligament, while somewhat greater concentrations were found in teeth with periapical radiolucencies of <1 cm diameter; finally, the greatest concentrations were measured in teeth with radiolucent lesions >1 cm, which confirms the relationship between TNF-*α* and the emergence and development of periapical lesions. Objective analysis of TNF-*α* levels enables establishment of a relationship between different concentrations of this cytokine and different radiological changes.
